# Multi-objective macro-geometry optimization of a compound planetary geartrain for an electric tractor powertrain using NSGA-II

**DOI:** 10.1038/s41598-026-43864-3

**Published:** 2026-04-17

**Authors:** Seung-Min Baek, Hyeon-Ho Jeon, Wan-Soo Kim, Yeon-Soo Kim, Yong-Joo Kim

**Affiliations:** 1 Eco-Friendly Hydrogen Electric Tractor & Agricultural Machinery Institute, Chungnam National University, Daejeon, 34134 Republic of Korea; 2 Department of Smart Agriculture Systems, Chungnam National University, Daejeon, 34134 Republic of Korea; 3 Department of Smart Bio-Industrial Mechanical Engineering, Kyungpook National University, Daegu, 41566 Republic of Korea; 4 Upland Field Machinery Research Center, Kyungpook National University, Daegu, 41566 Republic of Korea; 5 Department of Bio-Industrial Machinery Engineering, Pusan National University, Miryang, 50463 Republic of Korea; 6 Department of Smart Agriculture Systems Machinery Engineering, Chungnam National University, Daejeon, 34134 Republic of Korea

**Keywords:** Energy science and technology, Engineering

## Abstract

This study presents a multi-objective macro-geometry optimization of a compound planetary geartrain for an electric tractor powertrain. The proposed framework simultaneously minimizes peak-to-peak static transmission error and maximizes gear mesh efficiency under representative agricultural load conditions derived from load duration distribution data. The macro-geometry design variables include normal module, pressure angle, helix angle, and face width for two planetary gear sets integrated into a developed electric tractor prototype. A detailed geartrain model was developed using commercial analysis software, and the optimization was performed using the nondominated sorting genetic algorithm II with strength constraints based on ISO 6336 to ensure durability. The Pareto-optimal solutions were ranked using a criterion importance method based on variability and intercriteria correlation. Compared with the baseline prototype configuration, the optimized designs achieved a 14–16% reduction in transmission error across all gear pairs while maintaining or slightly improving mesh efficiency (up to + 0.16%p). The results demonstrate that macro-geometry refinement within fixed gear ratio and packaging constraints can effectively reduce excitation-related transmission error without compromising efficiency. Experimental validation of the optimized gear sets is planned in future work.

## Introduction

The global agricultural tractor industry is undergoing rapid transformation, with electric tractors emerging as an important segment in response to increasing demands for sustainable food production and reduced greenhouse gas emissions^1,2^. This transition is driven by rising fuel and labor costs, labor shortages in agriculture, and tightening environmental regulations^3,4^. Major agricultural machinery manufacturers have introduced battery-electric and hybrid-electric tractor platforms; however, challenges remain in improving drivetrain efficiency, durability, and vibration characteristics under high-load agricultural operating conditions. Electric tractor performance depends strongly on motor configuration. Current developments include single-, dual-, and multi-motor systems^5,6^. Among these, dual-motor architectures with separate traction and PTO motors enable flexible power summation and separation through planetary gear transmissions, maintaining high efficiency across diverse operating modes^7,8^.

In parallel, research on electric tractor design and optimization has expanded, with increasing use of advanced multi-objective optimization (MOO) algorithms to address trade-offs in mechanical systems^9,10^. Genetic algorithms, particularly the nondominated sorting genetic algorithm II (NSGA-II), have performed well in exploring complex design spaces, making them suitable for macro-geometry optimization of gear transmission^11^. Prior studies have primarily addressed optimization at the system level, focusing on improving specifications such as overall efficiency, traction capability, and energy management strategies, often under simplified or standardized load conditions^12,13^. Several scholars have investigated motor–gear matching, control algorithms, and planetary gear transmission architectures in general to enhance drivetrain performance^14^.

In previous studies, researchers have applied multiobjective optimization techniques, particularly genetic algorithms, to various aspects of electric tractor design ^15^. Zhang et al.^16^ optimized the gear ratios and battery capacities of a dual-motor drive system using a parameter optimization method based on driving cycles (POMBDC), reducing energy consumption by 3–11% compared with that of conventional configurations. Liu et al.^17^ employed NSGA-II to minimize traction motor thermal losses while maximizing total drivetrain efficiency, achieving a maximum 2% improvement in system efficiency and a 1.4% increase at the rated load. In the context of noise reduction, Choi et al.^18^ applied gear macro-geometry optimization using genetic algorithms to conventional tractor transmissions, reducing gear whine noise levels by approximately 3.1 dBA under the tested conditions. Cianciotta et al.^19^ implemented similar NSGA-II-based approaches in automotive e-axle gear design to improve mesh efficiency and minimize vibration excitation. While these studies have contributed to improving system-level performance, energy efficiency, and, in some cases, noise characteristics, most researchers have focused either on the overall powertrain or on fixed-axis gear pairs, without specifically addressing the compound planetary geartrain (CPGT) that underpins dual-motor power-split architectures in electric tractors. Moreover, few scholars have simultaneously optimized the peak-to-peak static transmission error (PPSTE) and gear mesh efficiency (GME) under realistic agricultural workload conditions remains a critical research gap, particularly in electric tractors where the absence of engine masking noise makes gear meshing phenomena more perceptible to operators Transmission error (TE) is widely recognized as the primary excitation source associated with gear whine^20^. Although the radiated noise level also depends on system dynamics and housing characteristics, reducing TE effectively decreases the excitation input to the drivetrain structure, thereby contributing to improved acoustic performance^21,22^.

Accordingly, in the present study, MOO is applied to the macro-geometry of a CPGT in an electric tractor powertrain, with the dual aims of reducing PPSTE and improving GME. The optimization framework incorporates geometric constraints, contact ratio limits, and strength criteria for bending and contact stresses to ensure both durability and feasibility. The optimized designs are evaluated using workload data derived from load duration distribution (LDD) under representative agricultural operations, enabling practical assessment of their potential to reduce transmission-error-related excitation while maintaining high GME. By integrating criterion importance through the CRITIC method for final design selection, a robust and transferable methodology is provided for balancing TE and GME in next-generation electric tractor drivetrains. To demonstrate this framework, the CPGT configuration and modeling approach are first presented, followed by the formulation of PPSTE, GME, and strength constraints. The MOO procedure and macro-geometry design variable settings are then introduced, and the resulting Pareto fronts are analyzed using CRITIC to identify balanced CPGT configurations.

## Methods

### Electric tractor powertrain

**Fig. 1:**
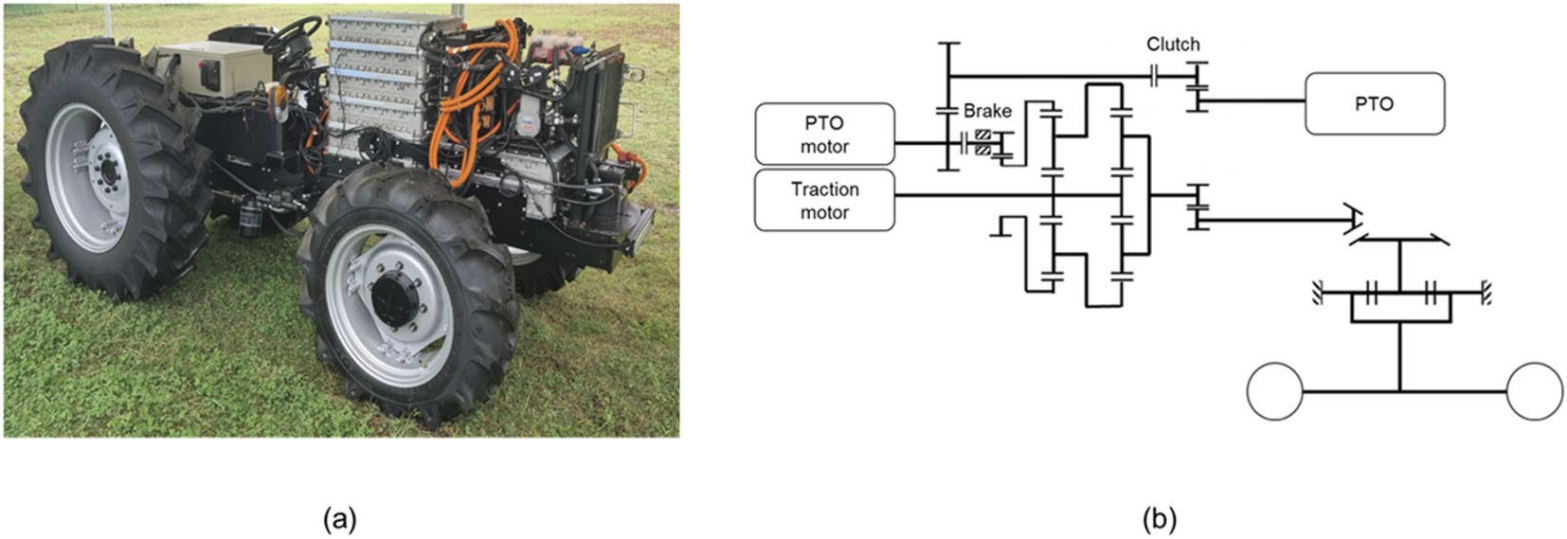
Electric tractor prototype and powertrain configuration incorporating a dual-motor system and a CPGT: (**a**) developed electric tractor prototype, (**b**) schematic layout of the power transmission system.

The developed electric tractor prototype is shown in Fig. 1a. The tractor integrates a dual-motor electric powertrain consisting of a main PTO motor and a traction motor. The layout of the power transmission system is illustrated schematically in Fig. 1b. The PTO motor is rated at 55 kW and delivers 240 Nm at 2,200 rpm, whereas the traction motor is rated at 20 kW with 87 Nm at 2,200 rpm. As shown in Fig. 1b, both motors are integrated through a CPGT, enabling a wide effective gear ratio range within a compact coaxial drivetrain layout. This configuration is particularly advantageous for agricultural tractors that must decouple vehicle travel speed from implement operating speed. By enabling independent control of traction and PTO power paths, the architecture allows stable implement operation while maintaining appropriate ground speed, which is essential for field tasks such as plowing and rotary tillage.

**Fig. 2:**
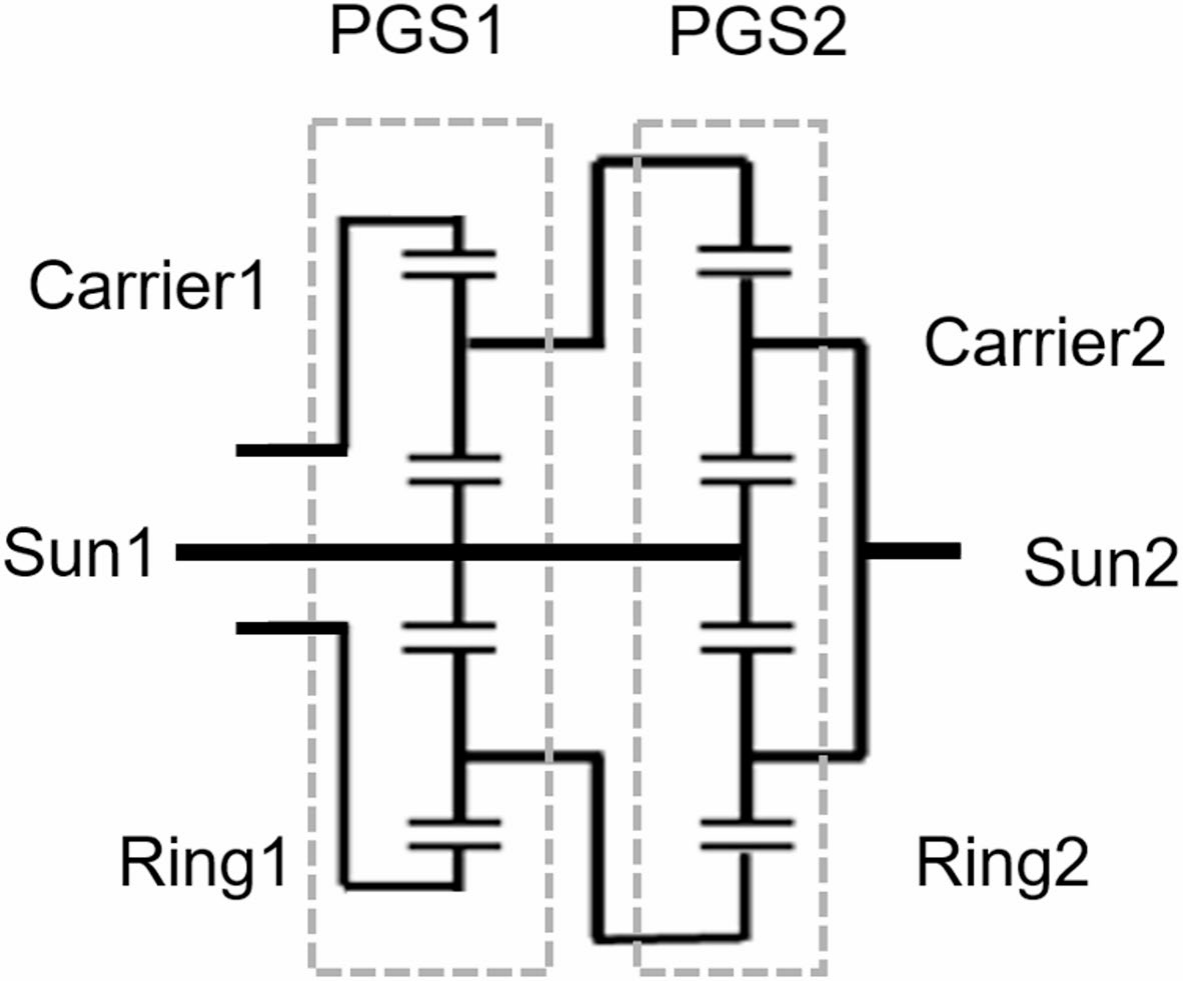
Configuration of the CPGT composed of two planetary gear sets.

The CPGT allows power summation and separation between the PTO and traction motors without the need for additional range-shift gears, thereby improving packaging efficiency and operational flexibility. In this configuration, the PTO motor is connected to the ring gear of PGS1, and the traction motor is connected to the sun gear of PGS1. The carrier of PGS1 is coaxially connected to the ring gear of PGS2, while the sun gear of PGS1 is linked to the sun gear of PGS2. The final output torque is transmitted through the carrier of PGS2 to the driving axle. Depending on operational requirements, the drivetrain operates in either power summation mode or power split mode, enabling flexible load sharing between the two motors. In the power summation mode, the PGS1 ring gear is mechanically constrained through the clutch and brake mechanism located upstream of PGS1. Under this condition, torque from both the PTO motor and the traction motor is coupled through the compound planetary linkage and merged at the PGS2 carrier, from which the combined torque is transmitted to the driving axle. This configuration enables direct torque summation before final output. In the power split mode, the constraint state is altered such that the torque paths are separated. The PTO motor delivers power directly to the PTO shaft, while the traction motor transmits torque to the axle through the compound planetary mechanism. In this mode, the planetary gear sets redistribute torque according to their kinematic relationships, allowing independent delivery of PTO and traction power within the same compact drivetrain architecture. The detailed configuration of the CPGT used in the prototype powertrain is presented in Fig. 2. As the key torque-coupling and ratio-adjustment component within the drivetrain, the CPGT directly influences transmission efficiency, load distribution, and static transmission error characteristics. Therefore, the present study focuses on the macro-geometry optimization of the CPGT implemented in the prototype configuration, while maintaining the same functional architecture and strength constraints.

### Geartrain design

The geartrain design process involves determining the optimal gear design by considering various factors to improve the performance of the gear system. This process is performed mainly to achieve the following goals: (1) increased GME, (2) satisfied durability, and (3) minimized PPSTE.

Noise performance was evaluated using the TE, which quantifies deviations from the ideal constant angular velocity transfer between gears. The PPSTE was calculated from the difference between the maximum and minimum TEs during a single mesh cycle. The TE can be expressed either as an angular displacement error between the driving and driven shafts or as a corresponding linear displacement along the line of action (LOA). The relationships for PPSTE, angular displacement, angular TE, and linear TE are given in Equations (1)–(4).

1 $$PPSTE = TE_{\max } - TE_{\min }$$

2 $$\theta_{p} r_{b\_p} = \theta_{w} r_{b\_w}$$

3 $$TE_{\theta } = \theta_{w} - \frac{{r_{b\_p} }}{{r_{b\_w} }}\theta_{p}$$

4 $$TE_{LOA} = \theta_{w} r_{b\_w} - \theta_{p} r_{b\_p}$$

where ${TE}_{\theta }$ is the transmission error, ${TE}_{LOA}$ is the transmission error at the LOA of the gear, $PPSTE$ is the peak-to-peak static transmission error, ${TE}_{max}$ is the maximum TE, ${TE}_{min}$ is the minimum TE, ${\theta }_{p}$ is the angular displacement of the pinion (rad), ${r}_{b\_p}$ is the radius of the base circle of the wheel (mm), ${\theta }_{w}$ is the angular displacement of the wheel (rad), and ${r}_{b\_w}$ is the radius of the base circle of the wheel (mm).

**Fig. 3:**
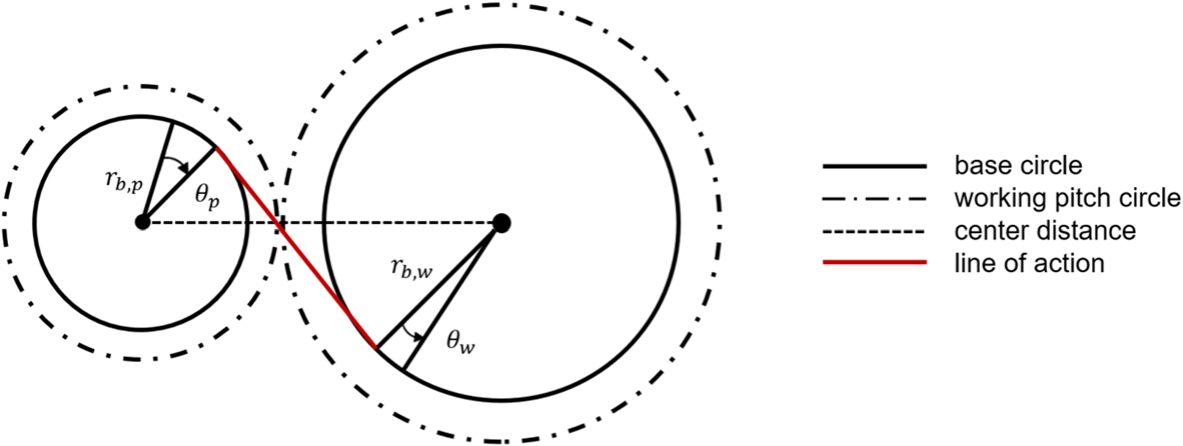
Geometric definition used for transmission error calculation.

The geometric relationships used in the TE computations are shown in Fig. 3. The base circle, pitch circle, and LOA define the reference geometry for Equations (2)–(4), allowing the conversion between angular and linear displacement errors. Linear displacement along the LOA is particularly useful for evaluating tooth shape modifications to reduce meshing impact, whereas angular displacement is more relevant for analyzing torsional vibration behavior.

The GME in this study is defined as the ratio of the output power to the input power of a gear pair, excluding losses associated with acceleration or deceleration of rotating components. This metric is critical for electric tractors because higher GME directly contributes to extended operating time per battery charge and reduced energy consumption during high-load agricultural operations. Following ISO/TR 14179-2 ^23^, the GME considering only load-dependent losses is expressed in Equation (5). It should be noted that the efficiency considered in this study represents GME only. Speed-dependent losses such as churning, windage, and seal friction were assumed constant across all candidate designs and were therefore not included in the optimization objective.

5 $$\eta = 1 - \frac{{P_{T2} }}{{P_{A} }}$$

where $\eta$ is the GME, ${P}_{T2}$ is the load-dependent mesh power loss as defined in ISO/TR 14,179–2, and ${P}_{A}$ is the applied power at the gear mesh.

Durability evaluation of the baseline and candidate designs was performed according to ISO 6336 Method B^24,25^. Both bending and contact stresses were calculated under maximum load conditions using the nominal stress formulations given in Equations (6)–(8). The resulting safety factors for bending and contact stresses were used to assess structural adequacy of each gear pair.

6 $$S_{F} = \frac{{\sigma_{FP} }}{{\sigma_{F} }},S_{H} = \frac{{\sigma_{HP} }}{{\sigma_{H} }}$$

where ${S}_{F}$ is the safety factor of bending stress, ${\sigma }_{FP}$ is the permissible bending stress (MPa), ${\sigma }_{F}$ is the tooth root stress (MPa), ${S}_{H}$ is the safety factor of contact stress, ${\sigma }_{HP}$ is the permissible contact stress (MPa), and ${\sigma }_{H}$ is the tooth contact stress (MPa).

7 $$\sigma_{F} = \sigma_{FO} K_{A} K_{V} K_{F\beta } K_{F\alpha }$$

8 $$\sigma_{H1,2} = Z_{B,D} \sigma_{HO} \sqrt {K_{A} K_{V} K_{H\beta } K_{H\alpha } }$$

where ${\sigma }_{F}$ is the tooth root stress (MPa), ${\sigma }_{FO}$ is the nominal tooth root stress (MPa), ${K}_{A}$ is the application factor, ${K}_{V}$ is the dynamic factor, ${K}_{F\beta }$ is the bending face load factor, and ${K}_{F\alpha }$ is the bending transverse load factor, ${\sigma }_{H\mathrm{1,2}}$ is the tooth contact stress of the pinion and wheel (MPa), ${Z}_{B,D}$ are the tooth contact factor of the pinion and wheel, ${\sigma }_{HO}$ is the nominal contact stress at the pitch point (MPa), ${K}_{A}$ is the application factor, ${K}_{V}$ is the dynamic factor, ${K}_{H\beta }$ is the contact face load factor, and ${K}_{H\alpha }$ is the transverse load factor.

### Simulation analysis

The baseline specifications of the CPGT are summarized in Table 1. The internal gear ratio of both PGS1 and PGS2 was set to 7.8, corresponding to a sun gear tooth number of 15 and a ring gear tooth number of 117. Three planet gears were employed in each PGS to ensure balanced load sharing and sufficient torque capacity. The baseline configuration utilized spur gears with a helix angle of 0°, a pressure angle of 20°, and modules of 3.0 mm for PGS1 and 3.5 mm for PGS2. The face widths were 20 mm for PGS1 and 30 mm for PGS2. These initial specifications were determined based on conventional gear design practice, prototype packaging constraints, target torque requirements of the electric tractor, and ISO 6336-based bending and contact strength criteria. Prior to optimization, the baseline design satisfied the required safety factors for bending and contact stresses, ensuring that it represented a strength-compliant and manufacturable configuration implemented in the developed prototype powertrain. The gears were made of SCM420H steel (density 7800 kg/m^3^), with a Young’s modulus of 207 GPa and a Poisson’s ratio of 0.30. Lubrication was provided by ISO VG 220 oil (density 870 kg/m^3^). The scuffing failure load stage was set to 12.

**Table 1:** Baseline specifications of the CPGT model used for macro-geometry optimization.

Item	Value					
	PGS1			PGS2		
	Sun	Planet	Ring	Sun	Planet	Ring
No. of teeth	15	51	117	15	51	117
No. of planet gear	3	3				
Module (mm)	3	3.5				
Helix angle (°)	0	0				
Pressure angle (°)	20	20				
Face width (mm)	20	30				
Material	SCM420H					
Lubrication	ISO VG 220					

**Fig. 4:**
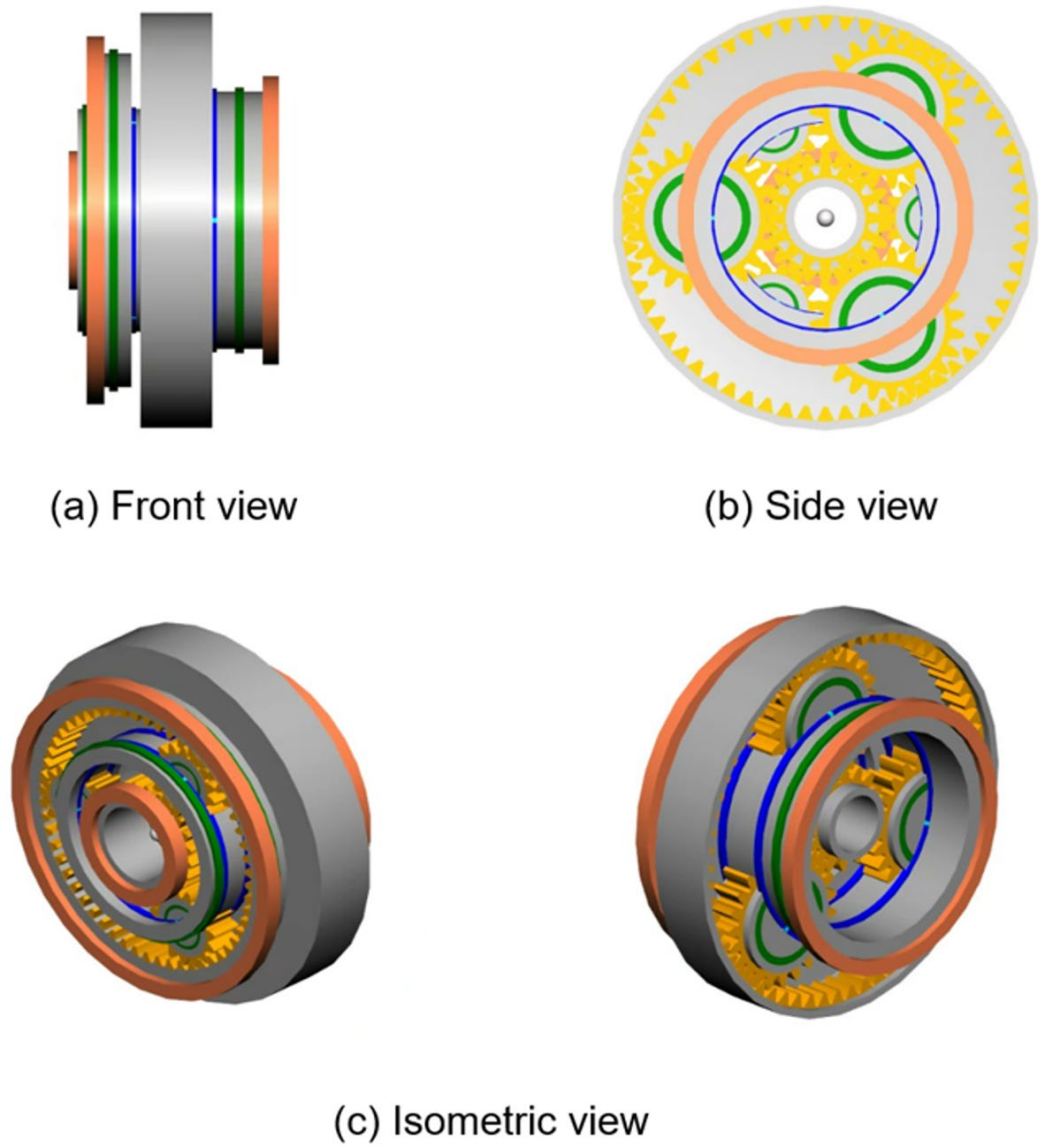
CPGT simulation model developed using MASTA: (**a**) front view, (**b**) side view, (**c**) isometric view.

The CPGT model was developed using MASTA (v13.0.1, Smart Manufacturing Technology Ltd., UK), an industry-standard gear analysis software implementing ISO 6336-based strength calculations and advanced tooth contact analysis. The software enables coupled modeling of compound planetary gear systems, ensuring proper kinematic relationships, torque equilibrium, and load sharing between the sun, planet, ring, and carrier members. Fig. 4 shows the CPGT simulation model configured with two planetary gear sets (PGS1 and PGS2) identical to the designed powertrain. The PGS1 carrier shaft and PGS2 ring gear shaft are integrated, while the PGS2 sun gear and carrier are coaxially aligned. Each PGS includes sun, ring, and planet gears, with the planet gears of PGS1 and PGS2 connected via a carrier component. The kinematic constraints between the sun, planet, ring, and carrier members of both planetary stages were explicitly defined to ensure correct compound linkage representation. The solver accounts for torque equilibrium and internal power circulation effects inherent to CPGT configurations. Six bearing models are incorporated: within the PGS1 ring gear shaft, PGS1 carrier shaft, PGS2 ring gear shaft, PGS2 carrier shaft, planet gear shaft, and sun gear shaft. Bearings linked to the PGS1 sun gear shaft, PGS1 carrier shaft, PGS2 ring gear shaft, and PGS2 carrier shaft are fixed to the housing via rigid constraints. For each macro-geometry candidate generated during the NSGA-II optimization process, the CPGT model was solved directly within MASTA, and the corresponding GME and PPSTE values were obtained from the coupled planetary gear solver. These solver-based outputs were used as the objective functions in the multi-objective optimization framework.

The simulation conditions of the CPGT model are summarized in Table 2. The load inputs were derived from the LDD data obtained using an agricultural tractor-based load measurement system (Agricultural Tractor for Load Measurement, NFEC-2025-06-306466). Eight representative load cases being assigned to each agricultural operation (plow tillage, rotary tillage, and driving), resulting in a total of 24 load cases^26^. A total annual working time of 342 h for a compact-utility tractor in Korea was applied over a service life of eight years, yielding a total duration of 2736 h^27^.

**Table 2:** Simulation conditions of the CPGT model in this study.

Load case	Operation	Torque (Nm)	Speed (rpm)	Duration (h)	Frequency (%)
1	Plow tillage	38.0	151.0	113.8	4.16
2		150.4	531.2	42.7	1.56
3		239.6	521.9	29.5	1.08
4		341.0	565.0	158.7	5.80
5		418.8	563.5	371.0	13.56
6		496.8	560.6	281.3	10.28
7		581.7	545.9	75.5	2.76
8		665.0	548.8	21.9	0.80
9	Rotary tillage	69.8	121.0	78.8	2.88
10		99.3	249.3	493.6	18.04
11		122.2	248.7	333.8	12.2
12		151.7	248.9	114.9	4.20
13		180.1	240.0	35.0	1.28
14		210.9	224.6	21.9	0.80
15		237.7	221.4	16.4	0.60
16		257.9	226.1	1.1	0.04
17	Driving operation	38.6	2304.6	331.6	12.12
18		57.7	2177.0	155.4	5.68
19		111.3	1850.4	17.5	0.64
20		153.3	1897	18.1	0.66
21		192.8	1145.6	8.8	0.32
22		242.8	512.2	8.8	0.32
23		270.7	388.4	4.9	0.18
24		316.2	462.7	1.1	0.04
Sum				2736.0	100.0

The operating ratios of plow tillage, rotary tillage, and driving were set to 4:4:2, with the duration for each operation being proportionally distributed (1094.4, 1094.4, and 547.2 h, respectively)^28^. Input torque was applied to the shaft of the PGS2 carrier, and the output was taken from the shaft of the PGS1 ring gear or sun gear, depending on the working mode of the powertrain.

### Multi-objective optimization

The macro-geometry of the CPGT was optimized using a MOO approach to simultaneously maximize gear durability and GME. Because these objectives often conflict, the NSGA-II was selected because of its ability to handle multiple objectives and generate Pareto-optimal solutions^29^

**Fig. 5:**
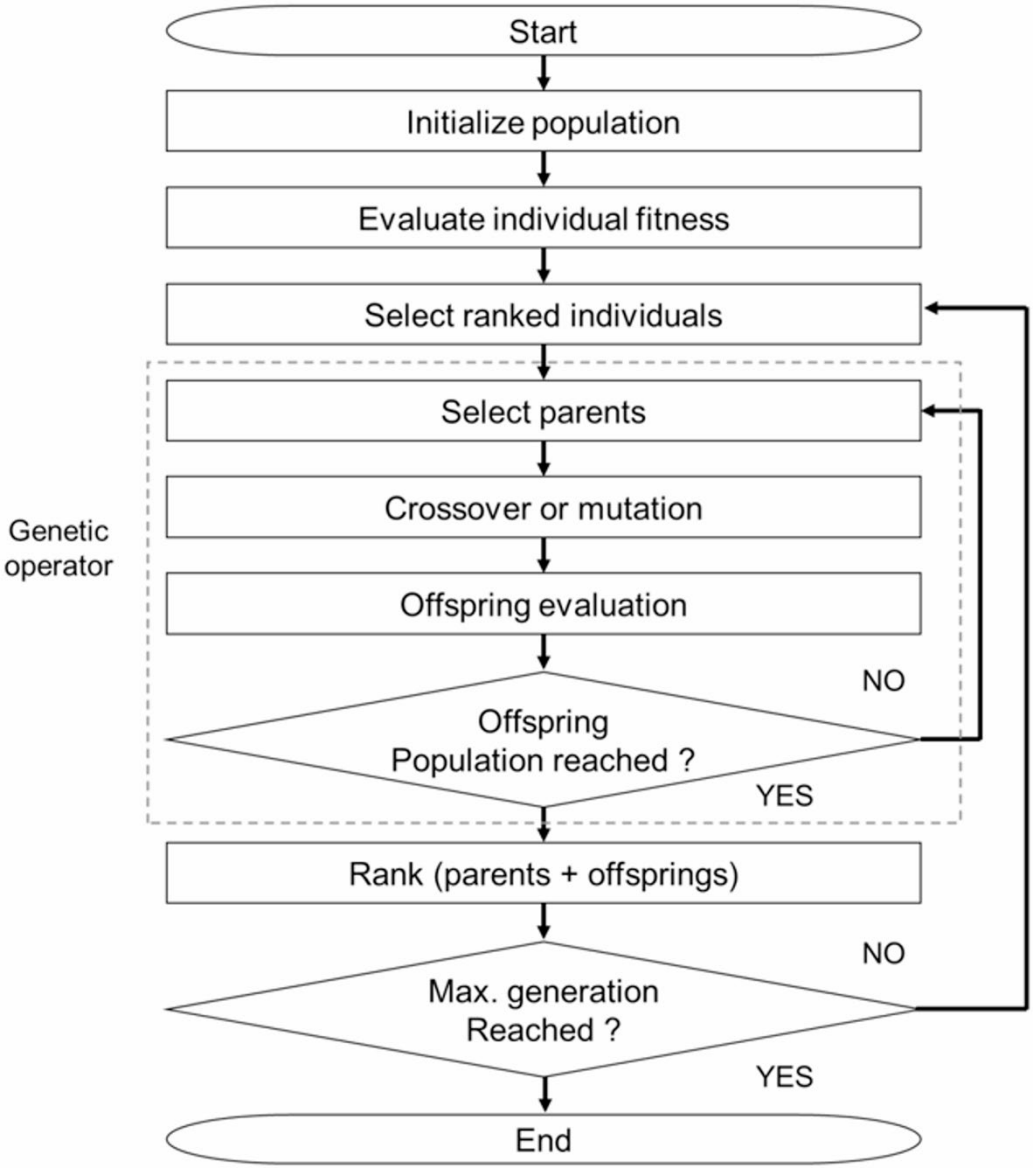
NSGA-II workflow for macro-geometry optimization of the CPGT.

As shown in Fig. 5, the NSGA-II process involved initializing a population of design candidates, evaluating their objective functions, and classifying them into nondominated fronts. Genetic operations, such as selection, crossover, and mutation, were then applied to create new generations, and the process was repeated until the convergence criterion or maximum generation was reached. This iterative approach enabled systematic exploration of the trade-off between durability and GME to identify optimal gear macro-geometry.

The macro-geometry design variables were the normal module, pressure angle, helix angle, and face width for both PGS1 and PGS2. The fundamental gear ratio of each PGS (7.8) and the corresponding gear tooth numbers were fixed according to the baseline prototype design, which was constrained by the predefined gearbox housing dimensions and kinematic layout. In the baseline configuration, the sun gear tooth number had been set to 15 for strength considerations, and the planet and ring gear tooth numbers were determined accordingly from geometric compatibility conditions. These tooth numbers remained unchanged throughout the optimization process and were not treated as design variables. Consequently, the optimization was strictly restricted to macro-geometry parameters while preserving the original gear tooth configuration and kinematic architecture. The normal module was determined from the fixed working center distance and the prescribed tooth numbers according to the standard geometric relationship for helical gears, as given in Equation (9).

Two objective functions of the MOO algorithm were used, namely, (a) minimization of the PPSTE and (b) maximization of the GME, as shown in Equations (10) and (11), respectively. Durability was set as a constraint because it had to be satisfied. PPSTE and GME were the representative performance metrics of the powertrains. Reduction in PPSTE implies a reduction in excitation-related transmission error. Higher GME implies improved power transmission capability under identical input conditions.

9 $$m_{n} = \frac{{2a_{w} \cos \beta }}{{Z_{s} + Z_{p} }}$$

where ${m}_{n}$ is the normal module (mm), ${a}_{w}$ is the working center distance (mm), $\beta$ is the helix angle of the gear pair ($^\circ$); and ${Z}_{s}$ and ${Z}_{p}$ are the numbers of teeth of the sun and planet gears, respectively.

10 $$PPSTE_{\min } = f_{PPSTE} \left( {m_{n} , \alpha , \beta , b} \right)$$

11 $$\eta_{\max } = f_{\eta } \left( {m_{n} , \alpha , \beta , b} \right)$$

where ${PPSTE}_{min}$ is the minimum PPSTE ($\mu$ m) and ${\eta }_{max}$ is the maximum GME (%), the macro-geometry design variables are the normal module ${m}_{n}$ (mm), pressure angle $\alpha$ (°), helix angle $\beta$ (°), and face width $b$ (mm).

The optimization constraints were defined to satisfy both the geometric and strength requirements of the CPGT, ensuring performance and durability. The contact ratio, expressed in Equations (12)–(14), represents the average number of tooth pairs engaged during meshing and influences load distribution and transmission smoothness. The aspect ratio, defined in Equation (15) as the ratio of the gear face width to the pinion base circle diameter, plays a key role in determining torque capacity, bending strength, and manufacturability. Based on Equation (16), the total contact ratio was constrained to 1.2–2.0, and the aspect ratio was restricted to the range of 1.0–2.0 to maintain adequate strength without excessive weight increase. The design variable ranges were determined based on prior studies and practical manufacturing guidelines provided by the collaborating gear manufacturer^30^. The resulting face width-to-diameter ratios remained within conventional engineering limits for hardened alloy steel gears.

12 $$\varepsilon_{\gamma } = \varepsilon_{\alpha } + \varepsilon_{\beta }$$

13 $$\varepsilon_{\alpha } = \frac{{g_{a} }}{{p_{b} }}$$

14 $$\varepsilon_{\beta } = \frac{b\sin \beta }{{\pi m_{n} }}$$

15 $$f_{b} = \frac{b}{{d_{1} }}$$

16 $$\left\{ {\begin{array}{*{20}c} {1.2 < \varepsilon_{\gamma } < 2} \\ {1 < f_{b} < 2} \\ \end{array} } \right.$$

where ${\upvarepsilon }_{\upgamma }$ is the total contact ratio, ${\varepsilon }_{\alpha }$ is the transverse contact ratio, ${\varepsilon }_{\beta }$ is the overlap ratio, ${g}_{a}$ is the length of the gear meshing on the LOA (mm), ${p}_{b}$ is the base circle pitch (mm), $b$ is the face width (mm), $\beta$ is the helix angle $(^\circ )$, ${m}_{n}$ is the normal module.

(mm), ${f}_{b}$ is the aspect ratio, and ${d}_{1}$ is the base circle diameter of the pinion (mm).

Gear strength was treated as a mandatory feasibility constraint in the MOO process. Only design candidates satisfying the minimum safety factor requirements were considered admissible solutions. Based on the ISO 6336-based stress evaluation described above, the safety factors were set to be no less than 1.2 for bending stress and 1.0 for contact stress to prevent pitting and tooth breakage as shown in Equation (17). These constraints were selected to guarantee sufficient load-carrying capacity and structural reliability under representative agricultural operating conditions.

17 $$\left\{ {\begin{array}{*{20}c} {S_{F} = \frac{{\sigma_{FP} }}{{\sigma_{F} }} \ge 1.2} \\ {S_{H} = \frac{{\sigma_{HP} }}{{\sigma_{H} }} \ge 1.0} \\ \end{array} } \right.$$

## Methodology

MOO was performed using the nondominated sorting genetic algorithm II (NSGA-II), which is suitable for problems involving multiple conflicting objectives. NSGA-II was selected due to its proven robustness and widespread application in multi-objective engineering optimization problems, rather than as a newly proposed optimization method. An automated CPGT simulation model was developed in Python, and macro-geometry optimization was conducted via the MASTA scripting function. To ensure sufficient convergence, the population size and number of generations were each set to 100, with crossover and mutation probabilities of 0.5. The gear ratio constant $k$ of the CPGT was fixed at 7.8. The design variables and their ranges are listed in Table 3. For PGS1 and PGS2, the normal modules were set to 2.5–3.5 mm and 3.0–4.0 mm, respectively; the pressure and helix angles were fixed at equal ranges of 15–25° and 0–30°, respectively; and the face widths were 20–25 mm and 30–35 mm, respectively. These ranges were determined on the basis of the CPGT and powertrain size constraints. Although the normal module and pressure angle are standardized in practical gear manufacturing, they were treated as continuous variables at the conceptual design stage to enable a comprehensive exploration of the macro-geometry design space. The optimized results therefore represent ideal parameter regions, which can be subsequently mapped to the nearest standardized values for practical implementation.

**Table 3:** Range of the design variable for the CPGT used in this study.

Item	Value			
	PGS1		PGS2	
	Lower	Upper	Lower	Upper
Normal module (mm)	2.5	3.5	3.0	4.0
Pressure angle (°)	15	25	15	25
Helix angle (°)	0	30	0	30
Face width (mm)	20	25	30	35

**Fig. 6:**
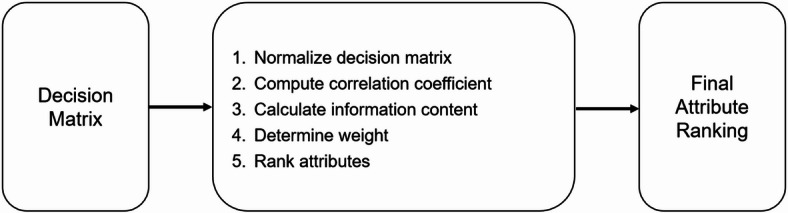
CRITIC-based decision-making process used to rank Pareto-optimal solutions.

In the final stage, the optimal solution was selected from the Pareto front using the criterion importance through the CRITIC method to determine the relative importance of each criterion on the basis of both the variability and correlation among the criteria^31^. The procedure comprised five steps: (1) normalizing the decision matrix, as shown in Equation 18; (2) computing correlation coefficients between criteria; (3) calculating the information content using both the correlation coefficient and standard deviation, as shown in Equation 19; (4) determining the weight factor for each criterion, as shown in Equation 20; and (5) ranking the attributes. The CRITIC weighting procedure was implemented according to the original formulation without modification. The overall workflow is illustrated in Fig. 6.

18 $$\overline{X}_{ij} = \frac{{X_{ij} - X_{j}^{worst.} }}{{X_{j}^{Best} - X_{j}^{Worst} }}$$

19 $$C_{j} = \sigma_{j} \times \mathop \sum \limits_{k = 1}^{m} \left( {1 - r_{jk} } \right)$$

20 $$W_{j} = \frac{{C_{j} }}{{\mathop \sum \nolimits_{k = 1}^{m} C_{j} }}$$

where ${\overline{X} }_{ij}$ is the normalized value of each criterion at the $i$ th, ${X}_{ij}$ is the value of each criterion at the $i$ th, ${X}_{j}^{Best}$ is the best value of each criterion, ${X}_{j}^{Worst}$ is the worst value of each criterion, ${C}_{j}$ is the amount of information, ${\sigma }_{j}$ is the standard deviation for each matching criterion, ${r}_{jk}$ is the correlation coefficient for each matching criterion, and ${W}_{j}$ is the weight factor for each matching criterion.

## Results

### Pareto front results

**Fig. 7:**
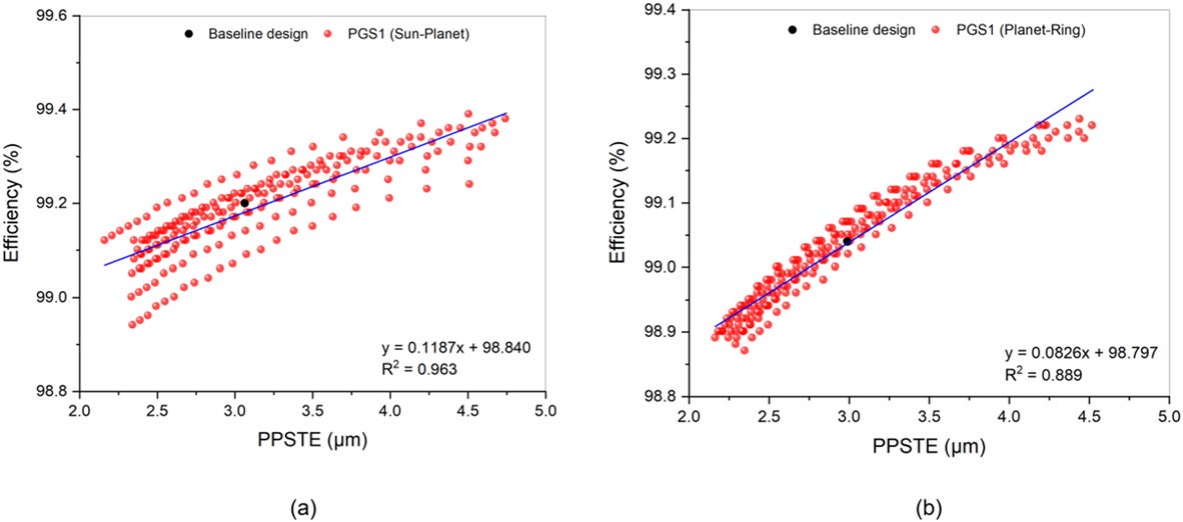
Pareto front results for PGS1: (**a**) sun–planet gear pair and (**b**) planet–ring gear pair.

The Pareto front results for PGS1 in the CPGT are shown in Fig. 7. For the sun–planet gear, the PPSTE ranged from 2.16 to 4.75 μm, and the GME ranged from 98.94% to 99.39%. For the planet–ring gear, the PPSTE ranged from 2.16 to 4.52 μm, with the GME ranging from 98.87% to 99.23%. The average PPSTE and GME were 3.15 μm and 99.19%, respectively, for the sun–planet gear and 3.01 μm and 99.04%, respectively, for the planet–ring gear.

A clear positive correlation was observed between PPSTE and GME in both gear pairs, as confirmed by regression analysis. The sun–planet gear exhibited a fitted slope of 0.1187 with an intercept of 98.840 (${R}^{2}$=0.963), indicating that the GME improved by approximately 0.45 percentage points across the full PPSTE range. The slope of the planet–ring gear was 0.0826, with an intercept of 98.797 (${R}^{2}=$ 0.889), and the GME increased by approximately 0.36 percentage points over its PPSTE range. These results demonstrated that configurations with slightly higher PPSTE tended to yield higher GME and that the strength of this relationship was greater for the sun–planet gear. The regression lines shown in the Pareto plots are provided solely as descriptive representations of the trade-off trend between PPSTE and GME within the obtained solution space. They are not intended as predictive models for other gear configurations.

This trend suggested that minimizing PPSTE alone did not necessarily yield maximum GME; instead, a balance between low transmission error and favorable meshing geometry was needed. A comparison of the two gear pairs revealed that the sun–planet gear was associated with not only a slightly higher maximum GME (99.39% vs. 99.23%) but also a wider PPSTE range, implying greater sensitivity to design variable changes. In contrast, the planet–ring gear yielded more stable PPSTE values, indicating lower variability in meshing performance. These observations could guide optimal macro-geometry selection by prioritizing gear pairs with stable PPSTE while achieving an GME that could exceed 99%.

**Fig. 8:**
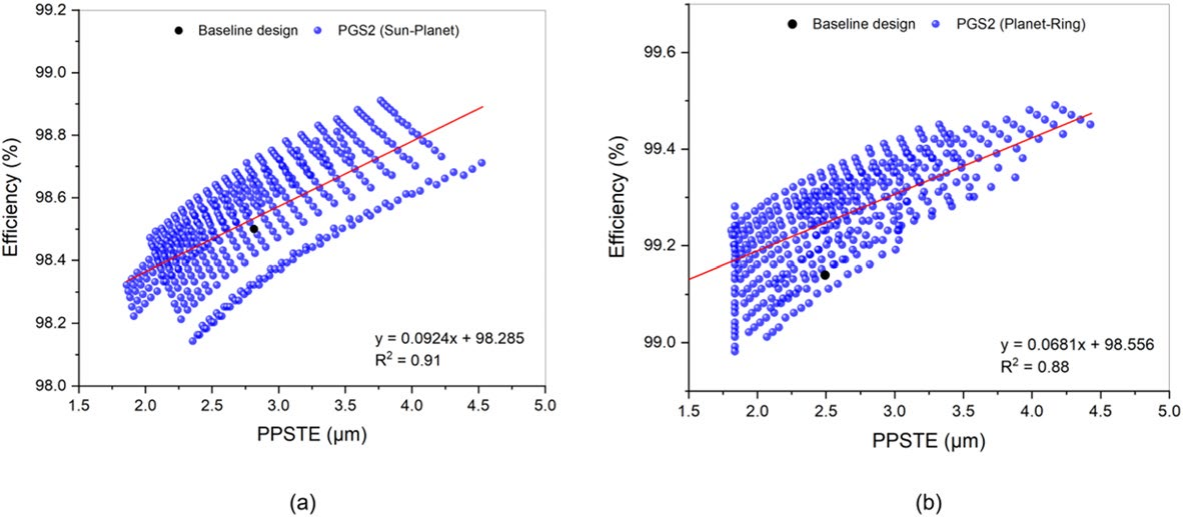
Pareto front results for PGS2: (**a**) sun–planet gear pair and (**b**) planet–ring gear pair.

The Pareto front results for PGS2 in the CPGT are shown in Fig. 8. For the sun–planet gear, the peak-to-peak static transmission error (PPSTE) ranged from 2.16 to 4.75 μm, and the GME ranged from 98.84% to 99.39%. For the planet–ring gear, the PPSTE ranged from 2.16 to 4.52 μm, with the GME ranging from 98.87% to 99.23%. The average PPSTE and GME were 2.82 μm and 98.53%, respectively, for the sun–planet gear and 2.46 μm and 99.24%, respectively, for the planet–ring gear.

Regression analysis confirmed positive correlations between PPSTE and GME for both gear pairs. For the sun–planet gear, the fitted slope was 0.0924, with an intercept of 98.285 (${R}^{2}$=0.91), corresponding to an GME increase of approximately 0.36%p across the PPSTE range. For the planet–ring gear, the smallest slope was observed among all the cases at 0.0681, with an intercept of 98.556 (${R}^{2}$=0.88), and the GME increased by approximately 0.25%p across its PPSTE range. The smaller slopes for PGS2 than for PGS1 indicated a reduced sensitivity of GME to PPSTE changes, especially in the planet–ring gear.

These results suggested that while moderate sensitivity and stable GME were observed for the sun–planet gear, the planet–ring gear was associated with the highest average GME and the smallest GME variation, making it the most robust configuration against changes in meshing geometry. For applications where stability and consistent performance were prioritized over maximum possible GME gains, the PGS2 planet–ring gear offered a clear advantage. The macro-geometry variables were applied simultaneously to both sun–planet and planet–ring meshes within each planetary stage, and their performance metrics were evaluated in a coupled manner to ensure mutual consistency.

The PGS1 and PGS2 configurations were compared in terms of GME potential and operational stability. PGS1 led to relatively sleep slopes in the PPSTE–GME relationship for both the sun–planet (0.1187) and planet–ring (0.0826) gears, indicating greater sensitivity of GME to changes in meshing geometry. This sensitivity led to PGS1, particularly the sun–planet gear, achieving increased peak efficiencies (up to 99.39%), and expanded the GME variation (0.45%p for the sun–planet gear).

In contrast, PGS2 was associated with lower slopes for both the sun–planet (0.0924) and planet–ring (0.0681) gears, indicating reduced sensitivity to PPSTE variation. This trend was the most pronounced in the PGS2 planet–ring gear, which achieved the highest average GME among all the cases (99.24%) while maintaining the smallest variation (0.25%). Such stability suggested that PGS2 configurations, especially the planet–ring gear, were highly suitable for applications requiring robust performance under variable load conditions or manufacturing tolerances. In summary, PGS1 was advantageous when the design objective prioritized maximum attainable GME, even at the expense of higher sensitivity to geometric changes. Conversely, PGS2 and its planet–ring gear configuration was preferable when the focus was on maintaining high GME with minimal performance fluctuation, offering a more robust solution for diverse operational scenarios.

### CRITIC-based ranking

Table 4 summarizes the CRITIC method results for both PGS1 and PGS2, which are derived from their respective Pareto front solutions. For each gear set, four criteria were considered: PPSTE of the sun–planet gear, PPSTE of the planet–ring gear, GME of the sun–planet gear, and GME of the planet–ring gear. The standard deviation (${\sigma }_{j}$​) of each criterion and the intercriteria correlation were used to compute the information content (${C}_{j}$​), followed by the calculation of normalized weights (${W}_{j}$).

**Table 4:** Results of the CRITIC-based weighting for the CPGT Pareto fronts.

Criterion			${\sigma }_{j}$	$\sum (1-{r}_{jk})$	${C}_{j}$	${W}_{j}$	Weight (%)
PGS1	PPSTE	Sun-Planet	0.247	3.83	0.947	0.24955	24.95
		Planet-Ring	0.255	3.78	0.963	0.2540	25.40
	GME	Sun-Planet	0.211	3.75	0.793	0.2090	20.90
		Planet-Ring	0.269	4.06	1.091	0.2875	28.75
	Total	-	-	3.793	1.0000	100.0	
PGS2	PPSTE	Sun-Planet	0.217	3.721	0.808	0.2657	26.57
		Planet-Ring	0.198	3.919	0.776	0.2553	25.53
	GME	Sun-Planet	0.219	3.468	0.758	0.2494	24.94
		Planet-Ring	0.205	3.412	0.698	0.2297	22.97
	Total	–	–	3.041	1.0000	100.0	

In PGS1, the planet–ring GME exhibited the highest weight (28.75%), followed by the planet–ring PPSTE (25.40%) and the sun–planet PPSTE (24.95%). In PGS2, the weighting distribution was more balanced, with all the criteria ranging between 22.97% and 26.57%, indicating a more uniform contribution of each criterion to the overall ranking. These weights were subsequently used to rank the Pareto front solutions in Tables 6 and 7.

Table 5 presents the ranked Pareto front solutions for PGS1, determined using the CRITIC method. The baseline values for the sun–planet gear were a PPSTE of 3.06 μm and an GME of 99.20%, whereas the planet–ring gear had a PPSTE of 2.99 μm and an GME of 99.04%. At Rank 1, the PPSTE of the sun–planet gear decreased to 2.57 μm (a 16.2% reduction compared with the baseline) while maintaining the same GME of 99.20%. The planet–ring gear at this rank showed a PPSTE of 2.56 μm, representing a 14.4% reduction, with the GME decreasing marginally by 0.1% to 98.99%. Across the top 10 ranked solutions, the sun–planet gear’s PPSTE values ranged from 2.50 to 3.37 μm, with efficiencies between 99.19% and 99.31%. The PPSTE of the planet–ring gear ranged from 2.49 to 3.36 μm, with efficiencies ranging from 98.97% to 99.14%. These results indicated that substantial reductions in PPSTE could be achieved without significant GME loss, particularly in the sun–planet gear. From a design standpoint, the rank 1 configuration offered the best trade-off between minimizing PPSTE and maintaining high GME. However, other high-ranking solutions (e.g., rank 2 and rank 10) showed that a small GME gain (reaching 0.11% for the sun–planet gear) could be obtained by allowing a slight increase in PPSTE, which could be acceptable depending on the operational priority between smoothness and peak performance.

**Table 5:** Results of the ranked Pareto fronts for PGS1 using CRITIC.

Rank	PGS1			
	Sun-Planet		Planet-Ring	
	PPSTE ($\mu m$)	GME (%)	PPSTE ($\mu m$)	GME (%)
Baseline	3.06	99.20	2.99	99.04
1	2.57	99.20	2.56	98.99
2	3.02	99.26	3.02	99.07
3	2.92	99.25	2.92	99.05
4	2.83	99.24	2.83	99.03
5	2.74	99.22	2.75	99.02
6	3.12	99.28	3.12	99.08
7	2.50	99.19	2.49	98.97
8	3.24	99.29	3.23	99.10
9	3.37	99.31	3.36	99.12
10	2.66	99.21	2.67	99.00

Table 6 presents a summary of the ranked Pareto front solutions for PGS2 derived using the CRITIC method. The initial values for the sun–planet gear were a PPSTE of 2.81 μm and an GME of 98.5%, whereas the planet–ring gear had a PPSTE of 2.49 μm and an GME of 99.14%. At Rank 1, the PPSTE of the sun–planet gear decreased to 2.39 μm, indicating a 15.1% reduction compared with that of the origin, whereas the GME increased slightly to 98.6% (+0.1%p). The PPSTE of the planet–ring gear decreased to 2.13 μm, indicating a 14.6% reduction, with the GME improving to 99.3% (+0.16%p). Across the top 10 ranked solutions, the PPSTE for the sun–planet gear ranged from 2.22 to 2.85 μm, with efficiencies between 98.54% and 98.73%. For the planet–ring gear, the PPSTE values varied from 1.98 to 2.54 μm, with efficiencies between 99.27% and 99.36%. This narrow GME range (<0.1%p) across multiple rankings confirmed the high stability of the PGS2 planet–ring gear, even when the PPSTE varied by more than 25% between solutions. From a design optimization perspective, the rank 1 configuration for PGS2 was associated with a substantial PPSTE reduction in both gear pairs without GME penalty. Particularly for the planet–ring gear, the GME remained consistently above 99.27% across all the top-ranked solutions, making it an ideal choice for applications where both smoothness (low PPSTE) and high GME would be critical.

**Table 6:** Results of the ranked Pareto fronts for PGS2 using CRITIC.

Rank	PGS2			
	Sun-Planet		Planet-Ring	
	PPSTE ($\mu m$)	GME (%)	PPSTE ($\mu m$)	GME (%)
Baseline	2.81	98.5	2.49	99.14
1	2.39	98.6	2.13	99.3
2	2.33	98.58	2.08	99.29
3	2.53	98.64	2.26	99.32
4	2.46	98.62	2.21	99.31
5	2.60	98.66	2.33	99.33
6	2.76	98.71	2.46	99.35
7	2.27	98.56	2.03	99.28
8	2.68	98.68	2.39	99.34
9	2.22	98.54	1.98	99.27
10	2.85	98.73	2.54	99.36

The optimal design analysis using the CRITIC method revealed distinct trade-offs between the PGS1 and PGS2 configurations. In PGS1, compared with that of the original solution, the rank 1 solution reduced the PPSTE of the sun–planet gear by 16.2% (3.06 → 2.57 μm) and that of the planet–ring gear by 14.4% (2.99 → 2.56 μm), whereas the GME was maintained within ±0.1%p of the baseline values. However, the GME variation across the top 10 ranked solutions in PGS1 was slightly greater—0.12%p for the sun–planet gear and 0.15%p for the planet–ring gear—reflecting increased sensitivity to geometry changes. In contrast, PGS2 yielded both lower PPSTE values and higher GME stabilities. The rank 1 configuration reduced the PPSTE by 15.1% for the sun–planet gear (2.81 → 2.39 μm) and by 14.6% for the planet–ring gear (2.49 → 2.12 μm), with the GME improving by a maximum of 0.16%p. Across the top 10 ranked solutions, the GME variation remained below 0.07%p for the planet–ring gear, indicating exceptional robustness even when PPSTE differences exceeded 25% between solutions.

Overall, while both PGS1 and PGS2 led to significant PPSTE reductions without sacrificing GME, PGS1 offered slightly higher peak efficiencies in some solutions, making it suitable for applications prioritizing maximum performance. However, PGS2—especially the planet–ring gear— superior GME stability and lower PPSTE across a wider range of configurations than PGS1, making it more appropriate for designs requiring consistent performance under variable manufacturing tolerances and operating conditions.

### Final optimal design

Table 7 presents a summary of the final specifications of the CPGT configurations selected through the NSGA-II optimization process. For PGS1, the sun–planet gear used a normal module of 3 mm, a pressure angle of 19.2°, a helix angle of 15.1°, a face width of 22.4 mm, and a center distance of 59.5 mm. The total contact ratio was 1.3555, with a transverse contact ratio of 1.1442 and an overlap contact ratio of 0.2113. The final PPSTE was 2.57 μm, which decreased from 3.06 μm (−16.2%), whereas the GME remained unchanged at 99.20%. The planet–ring gear shared the same macro-geometry, with a total contact ratio of 1.2448, a transverse contact ratio of 1.0335, and an overlap contact ratio of 0.2113. The PPSTE decreased from 2.99 μm to 2.56 μm (−14.4%), with only a marginal decrease in GME of 0.05% (99.04% → 98.99%).

**Table 7:** Results of the optimal solutions for the CPGT obtained using the NSGA-II.

Item	Value			
	PGS1		PGS2	
	Sun-Planet	Planet-Ring	Sun-Planet	Planet-Ring
Normal module (mm)	3		3.5	
Pressure angle (°)	19.2		24.1	
Helix angle (°)	15.1		15.4	
Face width (mm)	22.4		33.6	
Center distance (mm)	59.5		69.415	
Total contact ratio	1.3555	1.2448	1.6449	1.9055
Transverse contact ratio	1.1442	1.0335	1.3573	1.618
Overlap contact ratio	0.2113	0.2113	0.2876	0.2876
PPSTE ($\mu m$)	2.57	2.56	2.39	2.13
GME (%)	99.2	98.99	98.6	99.3

For PGS2, the sun–planet gear had a normal module of 3.5 mm, a pressure angle of 24.1°, a helix angle of 15.4°, a face width of 33.6 mm, and a center distance of 69.415 mm. The total contact ratio was 1.6449, with a transverse contact ratio of 1.3573 and an overlap contact ratio of 0.2876. The PPSTE decreased from 2.81 μm to 2.39 μm (−15.1%), whereas its GME increased slightly by 0.10% (98.50% → 98.60%). The PGS2 planet–ring gear had the highest contact ratios (total 1.9055, transverse 1.6180, overlap 0.2876), with the PPSTE decreasing from 2.49 μm to 2.13 μm (−14.6%) and the GME improving from 99.14% to 99.30% (+0.16%p).

Across all gear pairs, the optimized designs achieved a 14–16% reduction in PPSTE while maintaining or slightly improving GME. From a design selection perspective, PGS1 may be preferred when maximizing sun–planet GME is prioritized, whereas PGS2 provides a more robust configuration when minimizing transmission error and enhancing meshing stability are emphasized. The increased contact ratios observed in PGS2 contribute to smoother load transfer and reduced excitation levels associated with gear whine. It should be emphasized that the reported GME improvement refers specifically to GME. Speed-dependent no-load losses, such as churning and windage, were not included and were assumed identical across all configurations to ensure consistent comparative evaluation.

## Conclusions

In this study, a CPGT for an electric tractor powertrain was developed and optimized using an NSGA-II-based multi-objective optimization framework under representative agricultural workloads derived from LDD data. The optimization incorporated geometric constraints, contact ratio requirements, and ISO 6336-based strength criteria to ensure that all candidate solutions satisfied durability and feasibility conditions. PPSTE and GME were adopted as the primary evaluation metrics, representing transmission-error excitation characteristics and mesh-level power transmission performance.

The optimized configurations consistently reduced PPSTE by approximately 14–16% across all gear pairs while maintaining or slightly improving GME. The results demonstrate that coordinated adjustment of module, helix angle, pressure angle, and face width can effectively reduce transmission error without compromising mesh-level efficiency. From a practical design perspective, the proposed framework provides guidance for selecting macro-geometry parameters depending on whether GME -focused or PPSTE-focused performance is prioritized.

Several limitations should be acknowledged. The present study focused on macro-geometry optimization of the CPGT within the geometric and packaging constraints of the developed prototype gearbox. Because the overall gear ratio and housing dimensions were predetermined, the gear tooth numbers were fixed, and the design variables were therefore restricted to module, helix angle, pressure angle, and face width. Consequently, the optimization did not explore alternative tooth number combinations or ratio variations. Furthermore, speed-dependent no-load losses were not included in the efficiency objective; therefore, the reported improvements refer specifically to GME rather than total drivetrain efficiency. The findings are based on numerical simulations without experimental validation. Future work will involve manufacturing and experimentally validating the optimized gear sets and extending the framework toward broader design-variable exploration and integrated powertrain-level performance assessment.

## Data Availability

The datasets used and analyzed during the current study available from the corresponding author, [Y.J.K.], on reasonable request.
